# Within and between Whorls: Comparative Transcriptional Profiling of *Aquilegia* and *Arabidopsis*

**DOI:** 10.1371/journal.pone.0009735

**Published:** 2010-03-23

**Authors:** Claudia Voelckel, Justin O. Borevitz, Elena M. Kramer, Scott A. Hodges

**Affiliations:** 1 Allan Wilson Centre for Molecular Ecology and Evolution, Massey University, Palmerston North, New Zealand; 2 Ecology and Evolutionary Biology, University of Chicago, Chicago, Illinois, United States of America; 3 Department of Organismic and Evolutionary Biology, Harvard University, Cambridge, Massachusetts, United States of America; 4 Department of Ecology and Evolution, University of California Santa Barbara, Santa Barbara, California, United States of America; United States Department of Agriculture, Agricultural Research Service, United States of America

## Abstract

**Background:**

The genus *Aquilegia* is an emerging model system in plant evolutionary biology predominantly because of its wide variation in floral traits and associated floral ecology. The anatomy of the *Aquilegia* flower is also very distinct. There are two whorls of petaloid organs, the outer whorl of sepals and the second whorl of petals that form nectar spurs, as well as a recently evolved fifth whorl of staminodia inserted between stamens and carpels.

**Methodology/Principal Findings:**

We designed an oligonucleotide microarray based on EST sequences from a mixed tissue, normalized cDNA library of an *A. formosa x A. pubescens* F2 population representing 17,246 unigenes. We then used this array to analyze floral gene expression in late pre-anthesis stage floral organs from a natural *A. formosa* population. In particular, we tested for gene expression patterns specific to each floral whorl and to combinations of whorls that correspond to traditional and modified ABC model groupings. Similar analyses were performed on gene expression data of *Arabidopsis thaliana* whorls previously obtained using the Ath1 gene chips (data available through The Arabidopsis Information Resource).

**Conclusions/Significance:**

Our comparative gene expression analyses suggest that 1) petaloid sepals and petals of *A. formosa* share gene expression patterns more than either have organ-specific patterns, 2) petals of *A. formosa* and *A. thaliana* may be independently derived, 3) staminodia express B and C genes similar to stamens but the staminodium genetic program has also converged on aspects of the carpel program and 4) staminodia have unique up-regulation of regulatory genes and genes that have been implicated with defense against microbial infection and herbivory. Our study also highlights the value of comparative gene expression profiling and the *Aquilegia* microarray in particular for the study of floral evolution and ecology.

## Introduction

Flowers intrigue us because of their great diversity of form, colour and smell. This diversity is largely thought to be the result of co-evolution between flowering plants and pollinators, which dates to the Cretaceous when flowering plants first arose [1]. A key aspect of understanding the evolution of floral diversity requires the identification of the underlying genes. For one aspect of floral form, the identity of floral organs, the ABC model has been developed. It states that combinations of three classes of regulatory genes specify the development of sepals (A genes), petals (A + B genes), stamens (B+C genes) and carpels (C genes) [2]. It has been suggested that, once evolved, these regulatory genes could be recruited to other organs and transform them into new floral whorls. For example, B genes are expressed throughout the sterile whorls of monocots and many magnoliid dicots [3], [4], [5] and, as predicted by the ABC model, the entire perianths of these taxa have similar appearances as opposed to clearly distinct sepals and petals. Thus broad expression of B genes in perianth organs has been inferred to be ancestral in flowering plants whereas restriction of B gene expression to an inner whorl of petals in *Arabidopsis* and other eudicots is considered to be derived [5]. The differential presence of petals is thought to have been driven by the deployment of B gene expression to different positions in the flower after petal identity initially evolved [6], although others have suggested that petals truly evolved multiple times but recruited similar genes to control their development [7].

Many studies that have sought to relate variation in the number and appearance of floral whorls to modifications of the ABC model have examined expression patterns of ABC genes themselves. Recently, expression studies have expanded to include the genes and pathways that the ABC genes regulate both directly and indirectly [8]. Previously such wider analyses of floral gene expression were limited to the eudicot model plant *A. thaliana* [9], [10]. However, the development of microarrays for emerging model plants has enabled global studies elsewhere in the angiosperm tree, e.g., in the eudicot *Gerbera hybrida* [11], [12] and, most recently, the basal angiosperm *Persea americana* [8]. Using these global approaches, it is possible to compare whorl-specific expression patterns with co-expression in the traditionally defined A (sepals+petals), B (petals+stamens) and C (stamens and carpels) domains for many genes and contrast these patterns across different angiosperm lineages.

Apart from drawing attention to many genes simultaneously, global studies of gene expression also provide data that allow novel predictions of biological function. In the context of floral gene expression, this means that not only can expression patterns inform us about genes that are potentially involved in or are markers of the formation of floral organs, but they may also help formulate hypotheses regarding the specific functions of these organs. Such predictions are achieved through gene ontology and gene set enrichment analyses [13]. Expression data are tested for differentially regulated gene sets, which are defined a priori. Gene sets can be based on ontological terms of biological function, molecular function and cellular compartmentalization (www.geneontology.org). Thus the expression patterns for genes likely to underlie floral traits such as colour, scent, defense, nectar production, cell shape and cell size, micro- and macrosporogenesis can be compared within and between angiosperm lineages and provide markers for possible common attributes.

**Figure 1:**
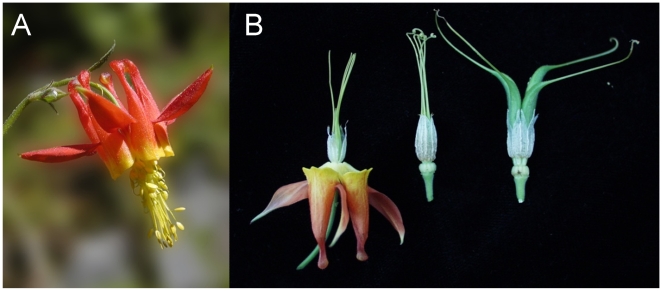
*A. formosa* pre-anthesis flower and fruit development. **A** *A. formosa* pre-anthesis flower. **B** Left: *A. formosa* pre-anthesis flower with stamens removed to expose staminodia. Middle and right: Early and later stages of fruit development, respectively. The sepals, petals and stamens dehisce while the staminodia remain attached to the receptacle and surround the carpels during fruit development.

The genus *Aquilegia* is a member of the Ranunculales, which is phylogenetically positioned as the first diverging branch of the eudicot clade (∼125 mya, [14]). The genus has undergone an adaptive radiation over the last two million years in North America into species that are primarily bee, hummingbird or hawkmoth pollinated and have corresponding morphological floral syndromes [15]. This floral diversity predisposes the genus as a model system for the investigation of pollinator-driven speciation [16]. The anatomy of the *Aquilegia* flower is also very distinct from many other angiosperm flowers. It has a bipartite perianth with petaloid sepals and petals that possess nectar-producing spurs, followed by four to seven whorls of stamens, one whorl of staminodia and one whorl of free carpels (Figure 1). Spurs and staminodia evolved only recently [17], [18] and while spurs are a modification of petals and produce a nectar reward for pollinators, the underlying developmental program and any specific function of staminodia are still a matter of debate. The sepals of *Aquilegia* are petaloid owing to their bright coloration and papillated epidermal cells [19]. However, it is not clear to what degree similar organ identity programs are operating in petals and petaloid sepals [19], [20]. Given these morphological features, the *Aquilegia* flower represents a particularly interesting case to study of the genetic basis of a) petaloidy, b) spur evolution and c) the recent evolution of a novel floral organ, the staminodia [20].

Growing interest from both the fields of floral genetics and adaptive radiation has prompted the development of a wealth of molecular resources for *Aquilegia* in the past years including a complete genome sequence [16]. Based on a normalized EST library generated from various tissues of an *A. formosa x A. pubescens* F2 population [20], a single channel oliognucleotide microarray platform representing more than 17,000 *Aquilegia* unigenes has been developed. Here we introduce this array and use it to obtain expression profiles from the five floral whorls of wild *A. formosa* flowers. In particular, we address questions such as: How distinct are the gene expression profiles of petals and petaloid sepals? Do petaloid sepals share expression patterns with petals and stamens (classic B-class organs)? To what extend do staminodia co-express genes with petals, stamens and carpels? Does the staminodia-specific gene expression profile suggest a possible ecological function to this novel organ? Can we identify candidate genes for the identity program for staminodia? To answer these questions, we investigate gene expression in individual whorls and groups of whorls in *A. formosa* and contrast our findings with those of a similar analysis on a publically available data set on the four floral whorls of *A. thaliana*. We then apply gene ontology analyses to identify biological processes operating in each whorl.

## Results

### Whorl-specific gene expression in *A. formosa* pre-anthesis flowers

#### Linear model analysis

**Figure 2:**
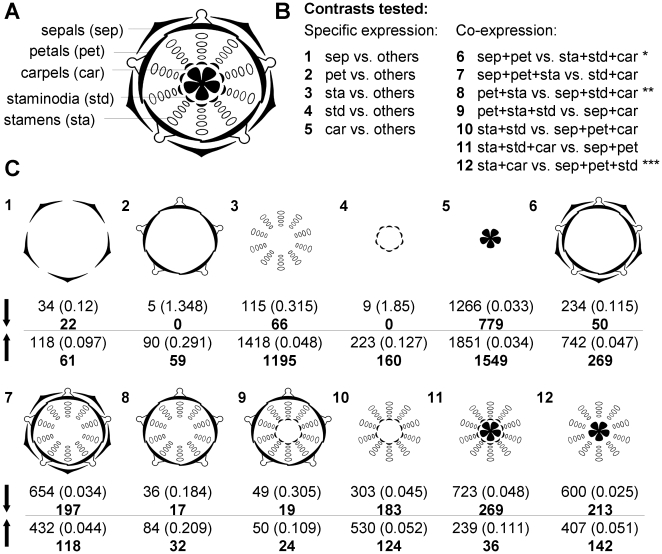
Differentially expressed genes in *Aquilegia* floral whorls and whorl combinations. **A** Floral diagram of an *Aquilegia* flower showing one whorl of five petaloid sepals, one whorl of five petals, four whorls of 10 stamens, one whorl of 10 staminodia and one whorl of five carpels. **B** Twelve contrasts were tested for differential expression, comparing each whorl against all others (1–5) and combinations of whorls against the remaining whorls (6–12). See text for details. **C** Numbers of down (downward arrow) and up (upward arrow) regulated genes for each of the 12 contrasts. First line shows number of differentially expressed genes with the corresponding permutation-based false discovery rate in brackets. Bold numbers below state the number of genes that have their highest absolute D statistic under that contrast and are being considered specifically expressed in the context of this study. Note that contrast 11 is reverse to contrast 6 and therefore yields similar numbers but with opposite regulation patterns.

We analysed gene expression in the five floral whorls of *Aquilegia formosa* late stage pre-anthesis flowers by fitting a linear model to expression data obtained with *Aquilegia* oligonucleotide arrays. Late stage pre-anthesis flowers are defined as the flower bud has opened, stamens have started to unfurl but anthers have not begun to dehisce [21]. Twelve different models were fit for each gene using different groupings of floral whorls, henceforth called contrasts. First, we tested the extent of whorl-specific expression (contrasts 1–5, Figure 2). Second, we tested for co-expression in sepals and petals, petals and stamens, and stamens and carpels (i.e., for genes expressed in the traditionally defined A, B and C domain, respectively; contrasts 6, 8, 12, Figure 2). Third, we tested groupings pertaining to the specific anatomy of the *Aquilegia* flower. Particularly, with contrast 7 we tested if the B domain is extended to petaloid sepals; with contrasts 9 and 11 we tested if the B and C domains are extended to staminodia and with contrasts 10 and 7 we tested the extent to which stamens and staminodia or carpels and staminodia were similar in gene expression, respectively. Numbers of up- and down-regulated genes for each contrast are summarized in Figure 2C. We also analysed whorl- and domain-specific gene expression patterns in two publically available datasets of *Arabidopsis thaliana*. These data are comprised of triplicate measurements of global gene expression in pre- and post-anthesis *A. thaliana* flowers (stage 12 and 15, [22]) obtained with Affymetrix Ath1 microarrays. The numbers of up- and down-regulated genes for seven *A. thaliana* contrasts examined in both floral stages and, for comparison, results for the corresponding seven *A. formosa* contrasts, are given in Figure S1. Lists of differentially expressed genes in *A. formosa* and *A. thaliana* can be found in Tables S2, S3, S4. In the following we describe expression patterns referring to specifically expressed genes as defined in Figure 2C.

During pre-anthesis in *A. formosa*, the majority of organ-specific gene expression was found in stamens and carpels whereas specific gene expression in sepals, petals and staminodia was comparably small (Figure 2C, Figure S1). In contrast, in *A. thaliana* pre-anthesis flowers (stage 12), stamens exhibited the largest extent of organ-specific gene expression followed by sepals, carpels and petals (Figure S1).

When considering sepals and petals combined, *A. formosa* had more genes specifically co-regulated (319) as compared to organ-specific gene expression (83 and 59 in sepals and petals respectively, Figure 2C). There were over four times as many genes co-up-regulated than specifically up-regulated in either of these organs (Figure 2C). In contrast, *A. thaliana* had fewer genes co-regulated (188 total) than those with organ-specific expression patterns (854 and 240 in sepals and petals respectively, Figure S1). These data reflect the petaloid nature of sepals in *A. formosa* compared to their distinct nature in *A. thaliana*. As expected due to the similar coloured nature of sepals and petals in *A. formosa*, homologues of four major genes of the anthocyanin biosynthetic pathway, namely chalcone synthase (TC14734), flavanone-3-hydroxylase (TC8210), dihydroflavonol-4-reductase (TC9974) and anthocyanidin synthase (TC18571), were all significantly co-up-regulated (Table 1) corroborating earlier findings [21]. Other genes co-regulated in sepals and petals may reflect other aspects of ‘petaloidy’.

**Table 1:** Expression statistics for a selected set of genes.

		D-statistics													Log intensities									
										B+	B+	STA+	C+		SEP		PET		STA		STD		CAR	
SEQ_ID	Annotation	SEP	PET	STA	STD	CAR	A	B	C	SEP	STD	STD	STD	# probes	m	se	m	se	m	se	m	se	m	se
***Anthocyanin Biosynthetic Pathway***																								
TC16794	Chalcone synthase	4.3	13.1	−3.9	0.7	−12.4	14.8	4.5	**−17.5**	6.4	5.6	−1.7	−13.7	24	**7.7**	0.1	**8.5**	0.2	6.7	0.1	**7.3**	0.1	5.9	0.0
TC14734	Chalcone synthase	9.1	5.3	−1.1	−9.7	−4.2	**13.8**	3.2	−2.0	10.3	−3.0	−6.3	−12.9	25	**10.0**	0.2	**9.7**	0.2	9.2	0.2	8.5	0.2	8.9	0.2
TC9292	Chalcone isomerase	5.2	4.2	**−16.3**	0.5	0.4	7.9	−4.6	−9.0	−0.3	−5.1	−8.6	−5.5	19	**9.3**	0.2	**9.2**	0.2	7.7	0.2	**8.8**	0.2	**8.8**	0.3
TC8210	Flavanone-3-hydroxylase	11.7	5.9	−8.3	−3.6	−4.7	20.0	−0.9	−10.7	6.8	−3.7	−7.8	**−20.1**	24	**10.3**	0.1	**9.9**	0.2	8.4	0.2	8.8	0.2	8.7	0.2
TC9974	Dihydroflavonol-4-reductase	12.8	9.1	−14.7	−2.7	−4.9	**29.1**	−2.0	−18.4	6.6	−4.0	−13.3	−19.6	25	**9.4**	0.1	**9.1**	0.2	5.8	0.1	7.2	0.2	6.9	0.2
TC18571	Anthocyanidin synthase	13.4	9.3	−11.2	−1.1	−7.7	26.7	−1.7	−20.2	7.5	−1.7	−10.0	**−32.2**	24	**10.3**	0.1	**9.8**	0.1	6.4	0.1	8.1	0.1	6.9	0.1
***B Genes***																								
TC17477	PISTILLATA-like protein	1.5	9.5	1.6	0.8	**−19.6**	8.3	8.8	−7.0	9.8	9.3	2.1	−6.6	21	**7.8**	0.3	**8.8**	0.3	**7.9**	0.3	**7.7**	0.3	5.8	0.1
TC11683	PISTILLATA-like protein	1.4	8.8	1.6	0.7	**−16.1**	5.8	8.0	−9.5	7.8	7.4	1.2	−7.1	22	**7.6**	0.3	**8.5**	0.3	**7.6**	0.3	**7.5**	0.3	5.8	0.1
TC11684	PISTILLATA-like protein	1.3	8.9	1.1	1.0	**−13.9**	7.5	7.4	−7.6	8.4	8.6	1.7	−6.7	23	**7.5**	0.3	**8.3**	0.4	**7.5**	0.3	**7.4**	0.3	5.8	0.1
TC19085	APETALA3-3	−4.5	**19.7**	1.2	−3.0	−5.1	6.0	13.3	−3.4	7.4	9.3	−1.3	−6.6	14	5.7	0.1	**8.9**	0.2	6.8	0.1	6.0	0.1	5.5	0.0
TC19725	APETALA3-2	−5.6	4.4	6.1	5.1	−12.5	−0.7	9.6	−3.2	3.8	**30.0**	8.3	0.7	15	6.6	0.1	**8.7**	0.2	**8.8**	0.2	**8.6**	0.2	5.8	0.1
TC16289	APETALA3-1	1.1	−3.5	2.3	**10.3**	−5.9	−1.6	−0.6	−5.2	0.2	6.3	9.8	1.6	19	7.4	0.2	6.8	0.2	7.6	0.3	**8.4**	0.3	6.0	0.1
***C gene***																								
TC8667	AGAMOUS-like protein	−8.7	−10.8	4.4	5.7	6.6	**−26.2**	−2.7	9.8	−9.2	1.2	7.9	17.8	23	6.3	0.2	6.1	0.2	9.0	0.2	9.0	0.3	9.2	0.3
***Transcription factors specifically expressed in staminodia***																								
TC13707	Myb	−4.9	−3.7	−4.8	**27.9**	−1.3	−6.0	−5.6	−4.3	−12.3	4.4	8.6	7.5	24	5.9	0.1	6.0	0.1	5.9	0.0	**8.8**	0.1	6.4	0.1
TC15349	Bzip	−2.5	−4.5	−3.4	**10.9**	0.1	−5.0	−6.9	−2.4	−9.7	1.6	4.3	4.9	26	6.6	0.2	6.4	0.2	6.5	0.2	**7.6**	0.3	6.8	0.2
TC15665	Heat shock	2.0	−1.2	−3.8	**11.2**	−5.6	0.4	−3.7	−7.4	−1.9	2.3	3.9	−0.4	19	6.4	0.2	6.1	0.1	5.8	0.1	**7.0**	0.3	5.6	0.1
TC16035	Homeobox 2 protein	0.3	1.1	−0.6	**11.5**	−11.3	1.1	0.0	−4.4	0.3	6.7	7.4	−1.2	26	7.3	0.1	7.4	0.1	7.2	0.1	**8.1**	0.2	6.5	0.1
TC17906	BEL1-related protein	2.8	−2.6	−1.5	**9.7**	−5.1	0.3	−3.6	−7.6	−0.7	1.9	4.2	−0.4	23	8.8	0.1	8.3	0.1	8.4	0.1	**9.2**	0.1	7.9	0.2
TC17508	BEL1-related protein	2.3	−2.5	−2.0	**11.9**	−8.9	0.2	−3.2	−6.1	−0.9	3.6	5.1	−0.2	25	8.2	0.2	7.8	0.2	7.8	0.2	**8.6**	0.2	7.4	0.2
TC15971	Knotted homeodomain	−0.8	−2.2	−1.4	**12.3**	−5.8	−2.0	−1.3	−6.1	−2.0	5.2	5.9	1.2	24	6.5	0.2	6.4	0.1	6.5	0.1	**7.4**	0.2	6.2	0.1

D statistics for anthocyanin biosynthesis genes, B genes, a C gene and transcription factors specifically expressed in staminodia are given under each of the 12 contrasts (figure 1, A = SEP+PET, B = PET+STA, C = STA+CAR) with the D statistic with the highest absolute value highlighted in bold. Also given are average log intensities per probe set and tissue (m) after quantile normalization including their standard errors (se) and numbers of probes in each set. The average log intensities corresponding to the contrast with the highest absolute D-statistic are highlighted.

Interestingly, petals and stamens combined (B domain) had few co-up-regulated genes (32 in *A. formosa* and 67 in *A. thaliana*) compared to petals and stamens separately (59 and 1195 in *A. formosa* and 214 and 1254 in *A. thaliana*, Figure S1). This pattern perhaps reflects the combination of B and C genes to determine stamen identity and the lack of C-gene expression in petals (at least in *A. thaliana*, [2]), in addition to the high transcriptional activity in stamens as opposed to petals in pre-anthesis flowers of both species. When we tested for coordinated expression in the B whorls along with either adjacent whorl in *Aquilegia*, fewer genes were co-up-regulated when staminodia were included (24) but nearly 4-fold more genes were co-up-regulated (118) when sepals were included (Figure 2C) suggesting a significant similarity of expression in sepals, petals and stamens after organ identity is established. These patterns are reflected to some extent by the expression of the B-class identity genes, *PISTILLATA* (*PI*) and *APETALA3* (*AP3*), in different ways. The *A. formosa* homologue of *PI* is represented by three probe sets (TC17477, TC11683, TC11684) which are all significantly co-up-regulated in sepals, petals, stamens and staminodia compared to carpels (Table 1). The up-regulation of *PI* in these four tissues has been demonstrated previously in *A. vulgaris* [19]. The expression of *PI* is thus extended to both sepals and staminodia in *Aquilegia*. The three *AP3* paralogues are expressed in a whorl-specific manner with *AP3-3* (TC19085) being most highly expressed in petals, *AP3-2* (TC19725) having the highest expression in petals, stamens and staminodia combined and *AP3-1* (TC16289) being most strongly expressed in staminodia (Table 1). Again, these patterns are consistent with the expression of the three *AP3* paralogues in *A. vulgaris* [19]. The expression of *AP3* paralogues is thus not extended significantly to sepals. However, the unique expression patterns of *AP3* paralogues in *Aquilegia* in petals, stamens and staminodia is hypothesized to contribute to the identity of these floral tissues [19].

Stamens and carpels (C domain) had far fewer co-up-regulated genes (142 and 50 in *A. formosa* and *A. thaliana* respectively) than stamens and carpels individually (1195 and 1549 in *A. formosa*; 1251 and 456 in *A. thaliana*, Figure S1). Again, this likely reflects the specific organ identity program of B and C genes for stamens and the lack of B-gene expression in carpels (at least for *A. thaliana*, [2]). When staminodia were included with the C whorls in *A. formosa* even fewer genes were co-up-regulated (36) (Figure 2C). Carpels and staminodia together co-up-regulated more genes (197 genes, Figure 2C, contrast 7; these genes are down-regulated in sepals, petals and stamens which is equal to up-regulation in staminodia and carpels) as compared to stamens and staminodia combined (124) (Figure 2C, contrast 10). Thus an interesting gene expression profile for staminodia emerges. In addition to the staminodia-specific up-regulation of 160 genes, there are 197 genes co-up-regulated with carpels and 124 genes co-up-regulated with stamens while stamens and carpels have 142 genes co-up-regulated (Figure 2C). These patterns are interesting given the fact that developmental, morphological and genetic evidence all suggest that staminodia are derived from stamens rather than carpels. However, the transcriptional similarity of staminodia and carpels may be due to shared morphological traits (e.g., staminodia and carpels are both laterally expanded laminar organs while stamens are not) rather than common ancestry. Support for the hypothesis that staminodia evolved from stamens as opposed to being an independently evolved whorl is that the *Aquilegia* homologue of the C gene *AG* (TC8667) is most highly up-regulated in stamens, staminodia and carpels combined while, as discussed above, the B genes *AP3-2* and *PI* are detected in both stamens and staminodia (Table 1). As the EST library from which our microarray was designed contained only one *AGAMOUS* gene (most similar to *AGAMOUS1* of *Aquilegia alpine*, *AqAG1*), no data are available for the expression of the second previously characterized locus, *AqAG2*, although studies indicate that this gene is carpel-specific [23]. Regulatory genes specifically up-regulated in staminodia include diverse transcription factors (myb, TC13707; Bzip, TC15349; heat shock, TC15665; homeobox 2, TC16035; BEL1-related, TC17906, TC17508; knotted, TC15971; Table 1).

#### Correlation analysis

Interestingly, in *A. formosa*, array-wide expression patterns were significantly negatively correlated between most whorls except for a significant positive correlation between petals and sepals and no significant correlation between sepals and staminodia (Table S1). In *A. thaliana*, array-wide expression patterns were significantly negatively correlated except for a positive correlation between petals and carpels (Table S1).

#### Gene set enrichment analysis

To test which, if any, biological processes were significantly up- or down-regulated in the whorls and whorl combinations of interest, gene ontology categories of biological processes (GOBP) commonly used to annotate *A. thaliana* loci were assigned to *Aquilegia* unigenes. A total of 2,571 *Aquilegia* unigenes were annotated with a total of 842 GOBPs. For the z-test of enrichment, we limited our test to GOBPs that had a minimum of 10 entries, reducing the number of *Aquilegia* unigenes and GOBPs to 2003 and 163, respectively. We used the z-test to determine if the mean D statistic of a GOBP differed from the mean D statistic of the 2003 genes of a given contrast. The significant GOBPs are listed in Table 2. GOBPs significantly up-regulated in sepals and petals separately and sepals and petals combined (the A domain) included ATP-dependent proteolysis and electron transport. Flavonoid biosynthesis genes were also up-regulated in the perianth, which corroborates earlier findings of up regulation of anthocyanin biosynthetic pathway genes in anthocyanin producing perianths of several *Aquilegia* species [21]. Another gene set up-regulated in the A domain involves genes responding to auxin stimulus. GOPBs specifically up-regulated in sepals and petals respectively were photosynthesis and aging. In line with expectations, stamens had significant up-regulation of genes involved in pollen development and pollen germination. GOPBs such as mitosis, cytokinesis, microtubule polymerization and movement, and vesicle-mediated transport indicate that *A. formosa* microspores undergo mitotic divisions involving phragmoblast-mediated cytokinesis. GOBPs significantly up-regulated in the B domain were dominated by those up-regulated in stamens, except for pollen germination and development and microtubule polymerization and movement which were down-regulated in petals. Gene sets enriched in carpels, such as gamete formation, DNA replication and nucleosome assembly indicate that during pre-anthesis, carpels prepare to form megaspores by synthesizing DNA prior to meiosis. Interestingly, stamens and carpels up-regulated different signalling pathways (GTPase mediated signal transduction in stamens, transmembrane receptor protein tyrosin kinase signalling pathway in carpels) and oppositely regulated photosynthesis (down-regulated in stamens and up-regulated in carpels). Both whorls combined (C domain) up-regulated additional categories such as DNA repair and regulation of progression through cell cycle. In staminodia, four GOBPs were significantly up-regulated, namely, lignin biosynthesis, response to wounding, fatty acid beta-oxidation and one carbon compound metabolic process, suggesting an important defence function of staminodia. None of the categories was shared with the four other whorls but similar to stamens, staminodia down-regulated photosynthesis.

**Table 2:** Significant GO categories.

GO ID	GO description	GO ID	GO description	GO ID	GO description
**sepals down**		GO.0007018	microtubule-based movement	GO.0009409	response to cold
GO.0006730	one-carbon compound metab.process	GO.0006012	galactose metabolic process	GO.0000226	microtubule cytoskeleton organization
GO.0045045	secretory pathway	GO.0007067	mitosis	GO.0007018	microtubule-based movement
GO.0007067	mitosis	GO.0007094	mitotic spindle checkpoint	GO.0051258	protein polymerization
GO.0000910	cytokinesis	GO.0006887	exocytosis	GO.0046785	microtubule polymerization
GO.0000074	regulation cell cycle progression	GO.0000082	G1/S transition of mitotic cell cycle	**A whorls up**	
GO.0006886	intracellular protein transport	GO.0007047	cell wall organization and biogenesis	GO.0006118	electron transport
GO.0007010	cytoskeleton organization/biogenesis	GO.0000226	microtubule cytoskeleton organization	GO.0006510	ATP-dependent proteolysis
GO.0006260	DNA replication	GO.0009826	unidimensional cell growth	GO.0008152	metabolic process
GO.0006412	translation	GO.0000160	two-component signal transduction	GO.0009813	flavonoid biosynthetic process
GO.0007169	transmembrane receptor protein	GO.0051301	cell division	GO.0009733	response to auxin stimulus
	tyrosine kinase signaling pathway	GO.0006888	ER to Golgi vesicle-mediated transport	GO.0006629	lipid metabolic process
GO.0007017	microtubule-based process			GO.0000162	tryptophan biosynthetic process
GO.0000226	microtubule cytoskeleton organization	**staminodia down**			
GO.0009826	unidimensional cell growth	GO.0006457	protein folding	**B whorls down**	
GO.0051258	protein polymerization	GO.0006779	porphyrin biosynthetic process	GO.0009908	flower development
GO.0046785	microtubule polymerization	GO.0015979	photosynthesis	GO.0009626	hypersensitive response
GO.0007018	microtubule-based movement	GO.0000162	tryptophan biosynthetic process	GO.0006397	mRNA processing
**sepals up**		GO.0006412	translation	GO.0000003	reproduction
GO.0006510	ATP-dependent proteolysis	**staminodia up**		GO.0006334	nucleosome assembly
GO.0009626	hypersensitive response	GO.0009809	lignin biosynthetic process	GO.0000398	nuclear mRNA splicing, spliceosome
GO.0015979	photosynthesis	GO.0009611	response to wounding	GO.0040007	growth
GO.0009695	jasmonic acid biosynthetic process	GO.0006635	fatty acid beta-oxidation	GO.0006412	translation
GO.0006118	electron transport	GO.0006730	one-carbon compound metab process	GO.0015979	photosynthesis
GO.0006812	cation transport	GO.0007568	aging	**B whorls up**	
GO.0009813	flavonoid biosynthetic process			GO.0006810	transport
GO.0006508	proteolysis	**carpels down**		GO.0006886	intracellular protein transport
		GO.0009225	nucleotide-sugar metabolic process	GO.0006099	tricarboxylic acid cycle
**petals down**		GO.0009738	abscisic acid mediated signaling	GO.0009225	nucleotide-sugar metabolic process
GO.0007010	cytoskeleton organization/biogenesis	GO.0009611	response to wounding	GO.0015031	protein transport
GO.0007059	chromosome segregation	GO.0006099	tricarboxylic acid cycle	GO.0007017	microtubule-based process
GO.0016192	vesicle-mediated transport	GO.0006118	electron transport	GO.0007264	small GTPase mediated signal trans.
GO.0009409	response to cold	GO.0006629	lipid metabolic process	GO.0009058	biosynthetic process
GO.0000226	microtubule cytoskeleton organization	GO.0008152	metabolic process	GO.0000910	cytokinesis
GO.0007018	microtubule-based movement	GO.0006635	fatty acid beta-oxidation	GO.0006897	endocytosis
GO.0051258	protein polymerization	GO.0006810	transport	GO.0000162	tryptophan biosynthetic process
GO.0046785	microtubule polymerization	GO.0007568	aging	GO.0007047	cell wall organization and biogenesis
GO.0009555	pollen development	**carpels up**		GO.0006839	mitochondrial transport
GO.0009846	pollen germination	GO.0006412	translation	GO.0006887	exocytosis
**petals up**		GO.0007169	transmembrane receptor protein	GO.0000160	two-component signal transduction
GO.0006118	electron transport		tyrosine kinase signaling pathway	GO.0006096	glycolysis
GO.0008152	metabolic process	GO.0015979	photosynthesis	GO.0006629	lipid metabolic process
GO.0009733	response to auxin stimulus	GO.0006260	DNA replication		
GO.0006510	ATP-dependent proteolysis	GO.0006334	nucleosome assembly	**C whorls down**	
GO.0007568	aging	GO.0000398	nuclear mRNA splicing, spliceosome	GO.0009809	lignin biosynthetic process
GO.0016575	histone deacetylation	GO.0040007	growth	GO.0009813	flavonoid biosynthetic process
GO.0000162	tryptophan biosynthetic process	GO.0007276	gamete generation	GO.0006508	proteolysis
GO.0009813	flavonoid biosynthetic process	GO.0006457	protein folding	GO.0006979	response to oxidative stress
GO.0006098	pentose-phosphate shunt	GO.0006364	rRNA processing	GO.0006510	ATP-dependent proteolysis
		GO.0006281	DNA repair	GO.0008152	metabolic process
**stamens down**		GO.0006414	translational elongation	GO.0006635	fatty acid beta-oxidation
GO.0015979	photosynthesis	GO.0000074	regulation cell cycle progression	GO.0006118	electron transport
GO.0006412	translation	GO.0006397	mRNA processing	GO.0009611	response to wounding
**stamens up**		GO.0000003	reproduction	GO.0007568	aging
GO.0006886	intracellular protein transport			**C whorls down**	
GO.0009225	nucleotide-sugar metabolic process	**A whorls down**		GO.0006412	translation
GO.0015031	protein transport	GO.0000074	regulation cell cycle progression	GO.0006260	DNA replication
GO.0009058	biosynthetic process	GO.0009555	pollen development	GO.0051258	protein polymerization
GO.0006944	membrane fusion	GO.0007067	mitosis	GO.0046785	microtubule polymerization
GO.0006810	transport	GO.0006412	translation	GO.0007018	microtubule-based movement
GO.0007264	small GTPase mediated signal trans.	GO.0006260	DNA replication	GO.0000074	regulation cell cycle progression
GO.0000910	cytokinesis	GO.0007059	chromosome segregation	GO.0000226	microtubule cytoskeleton organization
GO.0009846	pollen germination	GO.0007169	transmembrane receptor protein	GO.0006281	DNA repair
GO.0007017	microtubule-based process		tyrosinekinase signaling pathway	GO.0009826	unidimensional cell growth
GO.0006839	mitochondrial transport	GO.0007010	cytoskeleton organization/biogenesis	GO.0006334	nucleosome assembly
GO.0006099	tricarboxylic acid cycle	GO.0009826	unidimensional cell growth	GO.0007067	mitosis
GO.0016192	vesicle-mediated transport	GO.0007017	microtubule-based process	GO.0007169	transmembrane receptor protein
GO.0051258	protein polymerization	GO.0009846	pollen germination		tyrosine kinase signaling pathway
GO.0046785	microtubule polymerization	GO.0006886	intracellular protein transport	GO.0006275	regulation of DNA replication
GO.0009555	pollen development			GO.0000910	cytokinesis

Gene categories of biological processes down-regulated or up-regulated in the five floral whorls and traditional A, B and C whorl combinations of *Aquilegia formosa* pre-anthesis flowers. Significant gene ontologies (GO) were determined by gene set enrichment analysis and had Benjamini-Hochberg-adjusted p values<0.01.

### Correlation of floral expression regulation in potential *A. formosa – A. thaliana* homologues

When aligning a six frame translation of the 17,801 uni genes of the *Aquilegia* gene index with the *A. thaliana* proteome (TAIR 7), a match was found for 5,918 genes using BLASTx (E≤5E-06). Vice versa, for 13,511 *A. thaliana* proteins, a matching *Aquilegia* uni gene was identified using tBLASTn (E≤7E-06). An intersection of both queries resulted in 2,620 reciprocal pairs of *A. thaliana* proteins and *A. formosa* uni genes. For 2,000 of these, expression information was available from both the *Aquilegia* oligonucleotide array and the Ath1 array. To determine the extent of conservation in floral expression regulation between these potential *Aquilegia* – *Arabidopsis* homologues, their expression statistics (D statistics) were correlated. Rank-based correlation coefficients did not exceed 0.42 (Table 3). Gene expression in *A. formosa* sepals, stamens and carpels was most strongly, and significantly, correlated with their counterparts in the *Arabidopsis* flower. However, no significant correlation was found between the gene expression patterns of petals of both species, recapitulating their morphological differences on the transcription level. Instead, expression in *A. formosa* petals was significantly correlated with expression in *A. thaliana* sepals (0.27) and expression of *A. thaliana* petals was weakly correlated with expression in *A. formosa* carpels (0.11). Interestingly, expression in *A. formosa* staminodia was positively correlated with expression in *A. thaliana* stamens but negatively correlated with expression in *A. thaliana* carpels, lending support to the hypothesis that staminodia evolved from stamens rather than carpels. Correlations between whorl groupings were highest for A domains, followed by C and B domains (Table 3).

**Table 3:** Gene expression correlation between potential homologues of *A. formosa* and *A. thaliana*.

*A. formosa*	Sepals	Petals	Stamens	Staminodia	Carpels	A domain	B domain	C domain
*A. thaliana*								
Sepals	0.37*	0.27*	−0.19*	−0.09*	−0.19*			
Petals	−0.11*	0.03	−0.02	0.03	0.11*			
Stamens	−0.06	−0.18*	0.33*	0.16*	−0.23*			
Carpels	−0.25*	−0.07	−0.16*	−0.08*	0.42*			
A domain						0.34*	−0.01	−0.30*
B domain						−0.18*	0.20*	0.09*
C domain						−0.34*	0.01	0.30*

Spearman's rank correlation coefficients for correlations of D statistics of 2000 potentially homologous genes in pair-wise comparisons of *A. formosa* and *A. thaliana* (stage 12) floral whorls and traditional A, B, and C domains. Highest *A. formosa* correlation coefficients are given in bold whereas highest *A. thaliana* coefficients are italicized. Statistically significant coefficients are marked with an asterisk.

## Discussion

In this study we examined gene expression in the five floral whorls of *A. formosa* with a newly designed oligonucleotide microarray. One of our goals was to compare floral gene expression in the basal eudicot *A. formosa* with that in the core eudicot *A. thaliana*. Another aim was to identify genes co-expressed with floral identity genes and characterize the transcriptional signature of petaloid sepals. Lastly, we were interested in generating hypotheses regarding the evolution and ecological function of staminodia, a floral organ type recently evolved in *Aquilegia* and its close relatives *Semiaquilegia* and *Urophysa* [17], [18].

Our study demonstrated the utility and reliability of the *Aquilegia* microarray by validating previously obtained floral expression patterns. Particularly, the specific expression of anthocyanin biosynthetic genes in the A domain (sepals and petals), *AqAG1* in the C domain (stamens and carpels) and *PI* in all whorls except carpels as well as the unique expression of *AP3* paralogues in petals, stamens and staminodia corroborated earlier findings [19], [21].

When contrasting co-expression patterns between the lower eudicot *A. formosa* and the core eudicot *A. thaliana* we found that in the latter, organ-specific expression invariably exceeded co-expression between whorls (Figure S1, e.g., stage 12, sepals: 854, petals: 240, stamens: 1658, carpels: 558, A whorls: 188, B whorls: 186, C whorls: 188). In *Aquilegia* however, co-expression in the A domain (319) was considerably greater than in sepals (83) and petals (59) alone (Figure S1). Also, in *Aquilegia* co-expression between sepals, petals and stamens (305) was higher than expression in sepals (83) and petals (59) whereas in *Arabidopsis*, co-expression between sepals, petals and stamens (558) only exceeded expression in petals (240) (Figure S1). These patterns of co-expression are consistent with a similar comparative transcriptomics experiment of the basal angiosperm *Persea americana* and *A. thaliana* [8]. This study demonstrated domains of elevated floral gene expression extending across floral whorls in *Persea* as opposed to expression domains that were more constrained to individual whorls in *A. thaliana*. In particular, expression levels of *Persea* genes that clustered with *APETALA3* and *PISTILLATA* peaked in stamens but were also high in tepals and detectable in carpels [8]. In the case of *Persea*, the results could be interpreted in the context of the ‘fading borders’ model, which correlates the presence of morphological grades between floral organs with similar gradients of floral organ identity gene expression [24]. *Aquilegia* flowers do not have the same kind of morphological grades observed in magnoliid dicots but the presence of petaloid sepals and the stamen-derived staminodia may provide analogous patterns.

The expression patterns we observed suggest that petaloidy of *Aquilegia* sepals correlates with a high degree of co-expressed genes in petals and sepals (269 genes) as well as in sepals, petals and stamens (118 genes). Some of these co-expressed genes are likely to be involved in mediating aspects of petaloidy. For example, the production of floral pigments in sepals and petals is consistent with co-expression of anthocyanin genes in these organs. The other identified feature of petaloidy in *Aquilegia* is papillated epidermal cells [19]. However, we did not identify likely genes involved with this character, e.g., a *MIXTA* homolog [25], perhaps because they are expressed earlier in development than the pre-anthesis flowers we studied or the genes were not represented on our array. Developmental control of this feature of petaloidy is not determined by the expression of the B class gene *PISTILLATA* as its down regulation does not affect this character [16]. However, the relatively large number of co-expressed genes in petals and petaloid sepals suggest that while the genetic factors controlling organ identity at a higher level may differ between these organs, identity pathways converge on similar downstream effectors to produce similar coloration and cell types. Despite this common set of expressed genes in *Aquilegia* sepals and petals, expression of potentially homologous genes in the sepals of *Aquilegia* correlated most strongly with that of the sepals of *Arabidopsis* (Table 3). Thus, even though *Aquilegia* sepals are petaloid, they retain significant ‘sepaloid’ gene expression patterns as well.

Interestingly, we found no correlation in homologous gene expression in *A. formosa* and *A. thaliana* petals, which may be indicative of independent origins of petals in *A. formosa* and *A. thaliana* or may simply reflect their highly divergent morphologies. *Aquilegia* petals have been suggested to be derived from sterilized stamens [26] whereas an andropetaloid origin for *Arabidopsis* petals has recently been challenged and an bracteopetaloid origin has been suggested instead [27]. These two possible origins of petals – petaloid bracts vs sterilized stamens – were first discussed by Takhtajan [28]. On the transcriptional level, andropetals may have arisen through a repression of C gene expression in an outer whorl of stamens whereas bractopetals may have evolved by an expansion of B gene expression into pre-existing sterile organs [29]. Apart from independent evolution, the observed lack of correlation in homologous gene expression in *A. thaliana* and *A. formosa* petals may have resulted from a strong divergence of petal identity pathways during the divergence of the *Aquilegia* and the *Arabidopsis* lineage. The fact that homologous gene expression in *A. formosa* petals was most strongly correlated with that in *A. thaliana* sepals is most likely due to the significant convergence of expression patterns of *A. formosa* petals and sepals (Table S1) and the strong correlation of expression between *A. formosa* sepals and *A. thaliana* sepals.

The molecular mechanisms accompanying the evolution of new floral organs have often been investigated in the framework of the ABC model. For example, the lodicules of monocot grasses are hypothesized to be derived from petals because their identity is controlled homologues of the B genes *AP3* and *PI* (refs in [30]). Thus a novel identity program may have evolved through modifications to an existing identity program in the lodicule. In *Aquilegia*, following the stamen whorls, there is one whorl of staminodia that have been interpreted as being evolutionarily derived from fertile stamens due to similarities with the development of stamens [17]. The staminodia have a prominent central midrib with ruffled laminae extending to either side and unique epidermal cells. The laminae form an interlocking sheath around the developing ovary. Occasionally, anthers are observed on the tips of the staminodia (pers. obs.). Staminodia express both B and C class identity genes (*AG*, *PI* and the *AP3-1* paralog) in *A. vulgaris* [19] and *A. formosa* (this study), which is consistent with staminodia having evolved from stamens. We interpret our finding that staminodia co-expressed more genes with carpels than with stamens as being the result of convergence of gene expression due to similar morphological features of staminodia and carpels (e.g., their laminar nature) rather than a common evolutionary origin of both organs (Figure 2C).

Staminodia also displayed unique gene expression patterns. In pre-anthesis flowers, staminodia-specific gene expression exceeded that in sepals and petals. A set of approximately 160 genes was specifically up-regulated in staminodia including transcription factors that might be involved in regulating the expression of these genes or even in determining staminodium identity itself. Particularly interesting is the up-regulation of two potential *BEL1*-related loci (TC17906, TC17508) and one *Knotted* gene (TC15971) in staminodia. Proteins of both families have been shown to antagonistically interact with *AG* in the outer floral whorls [31]. However, BEL-Knotted complexes consisting of PENNYWISE (PNY), POUNDFOOLISH (PNF), SHOOT MERISTEMLESS (STM) and KNAT2 have been shown to positively interact with *AG* in the inner floral whorls [32]. Interestingly, the presence or absence of BEL1 in complexes containing AG-SEP3 is crucial for ovule and carpel identity, respectively [33]. When *BEL1* expression is missing, integuments are transformed into carpelloid tissue indicating the need for *BEL1* to promote ovule formation in the presence of *AG*. The fact that *BEL1*- and *Knotted*-related proteins are up-regulated in parallel with *AG* in staminodia of *A. formosa* leads us to suggest that these three proteins could potentially interact to affect staminodia-specific gene expression or even identity in *Aquilegia*. Analogously to antagonistic interactions in ovules [33] and outer floral whorls [31], *BEL1* and/or *Knotted* proteins might regulate *AG* (or complexes thereof) to repress its carpel identity function and enable staminodia identity instead. Clearly, additional experiments are necessary to test this hypothesis. For example, it would be interesting to conduct *in situ* hybridization experiments in early stages of floral meristem development. In addition, a virus-induced gene silencing system for transient knock-outs of gene expression has recently been established for *Aquilegia* [34] allowing us to manipulate the expression of all *Aquilegia* BEL1 and Knotted proteins.

Our gene set enrichment analysis also suggests a possible ecological function of staminodia. Since staminodia remain attached to the receptacle long after the other floral organs have abscised (Figure 1), one hypothesis is that staminodia are impregnated with herbivory defensive compounds that protect the early differentiating fruits [35]. Consistent with this hypothesis, we found lignin biosynthesis genes to be up-regulated in staminodia (e.g., ferulic acid-5-hydroxylase, TC12484, cinnamoyl-CoA reductase, TC8815, TC8816, caffeoyl-CoA-O-methyltransferase, TC11606, TC11605). A strong lignin barrier may protect the developing ovary from microbial and insect enzymes and thus confer protection against predation by pathogens [36] and herbivores. Among the 15 genes with the strongest staminodia-specific expression patterns were two laccases (TC17980, DT727506), two diphenol oxidases (TC12078, TC10815), a peroxidase (TC10188) and two repiratory burst oxidase genes (TC12632, TC13899). Interestingly, these classes of genes have been implicated in other systems for defense against microbial attack and herbivory due to their up-regulation in response to herbivory or mechanical wounding [37], [38], [39] and/or by specific counter defenses in herbivores [40]. Moreover, two phenylpropanoid biosynthesis genes were up-regulated in staminodia (phenylalanine ammonia lyase, TC10503, 4-coumarate-CoA ligase, TC12066, TC11075, TC10642). Phenylpropanoids are precursor not only in lignin biosynthesis but also for isoflavonoid phytoalexins which have been demonstrated to act in microbial defense [41]. Thus metabolomic analyses could augment our expression studies by further testing a defense and protection function for staminodia. Increased lignin production could also enforce the hydrophobic nature of staminodia and prevent excess moisture around the developing seeds. Along these lines, it would be particularly interesting to determine if removal of staminodia affects fruit development or damage.

In summary, our comparative microarray study has enabled a global perspective on floral gene expression in *A. formosa*. Not only were previous gene expression patterns confirmed but also transcriptional signatures of petaloidy were discerned and candidate genes for the regulation of staminodia-specific genes were identified. Using this newly designed microarray, further questions relating to special features of the *Aquilegia* flower such as spur formation can be addressed. For example, transcriptional patterns in spur-forming petals of *Aquilegia* species can be compared with those of spur-less petals in species of *Semiaquilegia*. Moreover, transcriptional signatures associated with different pollination syndromes may be obtained across the *Aquilegia* radiation to help characterize the genetics underlying pollinator-driven floral diversification.

## Materials and Methods

### EST library and microarray construction

An *Aquilegia formosa x pubescens* normalized cDNA library was constructed from mixed shoot and floral apical meristems, flower buds, leaves and roots from an F2 hybrid population (Invitrogen, USA). The sequencing of 50,000 clones by The Institute of Genomics Research (TIGR, Rockville, USA) led to 85,039 ESTs which assembled into 11,985 contigs (for which a tentative consensus sequence, TC, was obtained) and 5,816 singleton ESTs, resulting in transcribed sequence information for a total of 17,801 *Aquilegia* unigenes (The *Aquilegia* Gene Index, version 2.0, http://compbio.dfci.harvard.edu/cgi-bin/tgi/gimain.pl?gudb=aquilegia, for an analysis of release 2.1 refer to [20]). An isothermal set of oligonucleotide probes (3–35 probes per gene depending on length, Tm 76°C) were designed for 17,276 of these genes and used for microarray fabrication (NimbleGen Systems, Reykjavík, Iceland). A total of 17,246 *Aquilegia* uni genes were represented by more than three probes and therefore included in expression data analysis. The microarray platform specifics have been deposited at the Gene Expression Omnibus genomics data repository hosted by NCBI (GEO accession nr: GPL9791). Sequence variation between *A. formosa* and *A. pubescens* is very low [42], thus probes designed from *A. pubescens* specific alleles are expected to hybridize to *A. formosa* cDNA. Furthermore, comparisons of gene expression between *A. formosa* floral whorls will not be affected.

### Sampling

Three *Aquilegia formosa* populations growing in close proximity at Blue Canyon, Sonora Pass (Sierra Nevada mountains, CA) were sampled for this study. Sixty flowers, all from late pre-anthesis stage, were harvested from each population, dissected into the five floral whorls (sepals, petals, stamens, staminodia, carpels) and immediately frozen into liquid nitrogen. Total RNA was isolated (RNeasy kit, Qiagen, USA) from each tissue across the three replicate populations and 40ug of RNA were sent to NimbleGen Systems (Reykjavík, Iceland) for hybridization.

### Hybridizations to the *Aquilegia* oligonucleotide array

Cy3 labeled cDNA from the five tissues and the three biological replicates was singly hybridized to the *Aquilegia* oligonucleotide array and raw intensities can be found in fifteen files (90004_532.pair, stamens 2; 90005_532.pair, carpels 3; 90013_532.pair, sepals 3; 92391_532.pair, carpels 2; 92477_532.pair, staminodia 2; 92535_532.pair, petals 2; 95084_532.pair, sepals 1; 95191_532.pair, stamens 1; 95192_532.pair, sepals 2; 95195_532.pair, staminodia 1; 95198_532.pair, stamens 3; 98340_532.pair, carpels 1; 98348_532.pair, petals 1; 98350_532.pair, petals 3; 99928_532.pair, staminodia 3, GEO series record: GSE19432). Labelling, hybridization and scanning was performed by NimbleGen Systems Inc., Madison, WI USA, following their standard operating protocols (order number OID5089).

### Source of *Arabidopsis thaliana* microarray data

Gene expression data of stage 12 and 15 *A. thaliana* flowers were generated by the *Arabidopsis* gene expression atlas project. These data sets are part of a developmental series of floral expression data generated from experiments with *A. thaliana* Col-0 plants and they are available from NCBI's Gene Expression Omnibus (http://www.ncbi.nlm.nih.gov/geo/query/acc.cgi?acc=GSE5632, [43]). Triplicate expression data were retrieved for sepals, petals, stamens and carpels from stage 12 and stage 15 flowers [22]. The respective raw data files used in the analysis were: ATGE_34 wild type flowers stage 12, sepals (GSM131585.CEL, GSM131586.CEL, GSM131587.CEL), ATGE_35 wild type flowers stage 12, petals (GSM131588.CEL, GSM131589.CEL, GSM131590.CEL), ATGE_36 wild type flowers stage 12, stamens (GSM131591.CEL, GSM131592.CEL, GSM131593.CEL), ATGE_37 wild type flowers stage 12, carpels (GSM131594.CEL, GSM131595.CEL, GSM131596.CEL), ATGE_41 wild type flowers stage 15, sepals (GSM131603.CEL, GSM131604.CEL, GSM131605.CEL) ATGE_42 wild type flowers stage 15, petals (GSM131606.CEL, GSM131607.CEL, GSM131608.CEL), ATGE_43 wild type flowers stage 15, stamens (GSM131609.CEL, GSM131610.CEL, GSM131611.CEL), ATGE_45 wild type flowers stage 15, carpels (GSM131612.CEL, GSM131613.CEL, GSM131614.CEL). Data were obtained using Affymetrix GeneChip Arabidopsis ATH1 Genome Array (http://www.ncbi.nlm.nih.gov/geo/query/acc.cgi?acc=GPL198). For the purpose of this study only probes located in exons designed to the sense strand were analysed, which reduced the number of probes to 352002 representing a total of 17,246 genes (genes with less than 4 probes were omitted).

### Microarray analysis

#### *A. formosa*

Raw intensity data were log-transformed, spatially corrected [44] and quantile-normalized [45]. After correcting for probe effect (by subtracting probe means), the gene means were determined from each probe set. For each of the 17,246 genes, a linear model was fit either using individual tissues or combinations thereof as fixed effects (Figure 2B) and populations as random effects. The goal was to test how many genes were specifically expressed in each tissue (up- or down-regulated with respect to the other four) and how many genes would be co-expressed in 1) sepals and petals (the traditional A domain, contrast 6), 2) petals and stamens (the traditional B domain, contrast 8), 3) stamens and carpels (the traditional C domain, contrast 12), 4) sepals, petals and stamens (contrast 7) to test to what extend the B domain is extended to petaloid sepals, 5) petals, stamens and staminodia (contrast 9) to test the extension of the B domain to staminodia, 6) stamens and staminodia (contrast 10) and stamens and carpels (contrast 7) to test if gene expression in staminodia would be more similar to that in stamens or carpels. A linear model was fit to each gene in both the experimental data as well as 99 sets of permuted data with arrays re-sampled from the experimental dataset. A standardized expression value (D statistic = coefficient/ (error + median error of all genes), [46]) was then calculated for each gene in the experimental and the permuted data sets. Ranked D-statistics of the permuted data sets were averaged for each gene and compared to the D-statistics of the experimental dataset. False discovery rates were calculated using a delta threshold of 4. In Figure 2C, numbers of differentially expressed genes under each contrast are given with their corresponding false discovery rate. Some genes were significantly differentially expressed under more than one contrast. For the purpose of this study we define genes as being specifically expressed under a given contrast when the D statistic for that contrast is the highest absolute D statistic across all contrasts (bold numbers in Figure 2C).

#### *A. thaliana*

The dataset with the four floral whorls in stage 12 was separately analysed from the dataset with four floral whorls in stage 15. Normalization was performed as described for the *Aquilegia* arrays. Similarly to the *A. formosa* analysis, a linear model was fit for each gene using each of the four tissues and combinations thereof as fixed effects. The goal was to test how many genes were specifically expressed in each tissue (up- or down-regulated with respect to the other three) and how many genes would be co-expressed in 1) sepals and petals (the traditional A domain), 2) petals and stamens (the traditional B domain) and 3) stamens and carpels (the traditional C domain). Permutation based false discovery rates were calculated as described for *A. formosa*. Numbers of differentially expressed genes for both *A. thaliana* datasets are contrasted with those from *A. formosa* for the seven contrasts that were common to all three analyses in Figure S1. All normalization and permutation analyses were carried out using customized R-scripts which can be found at http://naturalvariation.org/aquilegia.

### Gene set enrichment analysis

Gene ontology matrices of biological processes were constructed for genes on both arrays. The *A. thaliana* matrix consisted of 25,111 gene loci annotated with 1,542 GO categories [47]. Only GO categories with at least 10 genes and only genes that were present on the Ath1 array were used in parametric gene set enrichment analysis. Applying these filters led to a final matrix of 16,985 genes annotated with 306 GO categories. In case of *Aquilegia*, 2132 GO categories were assigned to 5,889 *Aquilegia* unigenes through the Plant Gene Index project (http://compbio.dfci.harvard.edu/tgi/plant.html). An *Aquilegia* GO matrix was then designed by eliminating GO terms not related to plants and GO terms with less than ten genes resulting in a final matrix of 2003 genes annotated in 163 GO categories. Parametric analyses of gene set enrichment were performed on D statistics of both *A. thaliana* datasets and the *A. formosa* dataset based on statistical procedures described in [48]. Results from *A. thaliana* are not shown but results from *A. formosa* are summarized in Table 1.

### *Aquilegia*-*Arabidopsis* homology assignment

First, a six frame translation of the 17,801 unigenes of the *Aquilegia* Gene Index (AQGI.release_2) was aligned against the *A. thaliana* proteome (TAIR7_pep_20070425) using the BLASTx algorithm. Then, the *A. thaliana* proteome was matched against the six translations of the *Aquilegia* unigene set using tBLASTn. Good quality hits from both alignments were extracted based on E values. Entries found in both alignments represent reciprocal matches between *A. thaliana* and *Aquilegia* and were considered potential homologues [49] for the purpose of this study.

## Supporting Information

Figure S1 Differentially expressed genes in *A. formosa* (pre-anthesis) and *A. thaliana* (stages 12 and 15) flowers. Each square represents one contrast and reports the number of differentially genes, the corresponding false discovery rate as determined by bootstrap analysis in brackets and the number of differentially expressed genes adjusted for genes with higher D statistics with other contrasts. Upper and lower panel depict numbers for down- and up-regulated genes, respectively.
(5.74 MB TIF)

Table S1 Spearman's rank correlation coefficients of array-wide expression of all pair wise combinations of whorls (* denotes p<0.001). In *Aquilegia formosa* (AF), most correlations are negative, except for a positive correlation of sepals and petals and no correlation between staminodia and sepals. In *Arabidopsis thaliana* (AT, stage 12), most correlations are also negative, except for a positive correlation between carpels and petals.
(0.05 MB DOC)

Table S2 Differentially expressed genes in late pre-anthesis *A. formosa* floral whorls.
(5.15 MB XLS)

Table S3 Differentially expressed genes in stage 12 *A. thaliana* floral whorls.
(1.57 MB XLS)

Table S4 Differentially expressed genes in stage 15 *A. thaliana* floral whorls.
(1.65 MB XLS)
