# Supplementary figures and images for: Within and between Whorls: Comparative Transcriptional Profiling of Aquilegia and Arabidopsis
Source: PLoS One. 2010 Mar 23;5(3):e9735. doi: 10.1371/journal.pone.0009735 (PMC2843724; doi:10.1371/journal.pone.0009735)

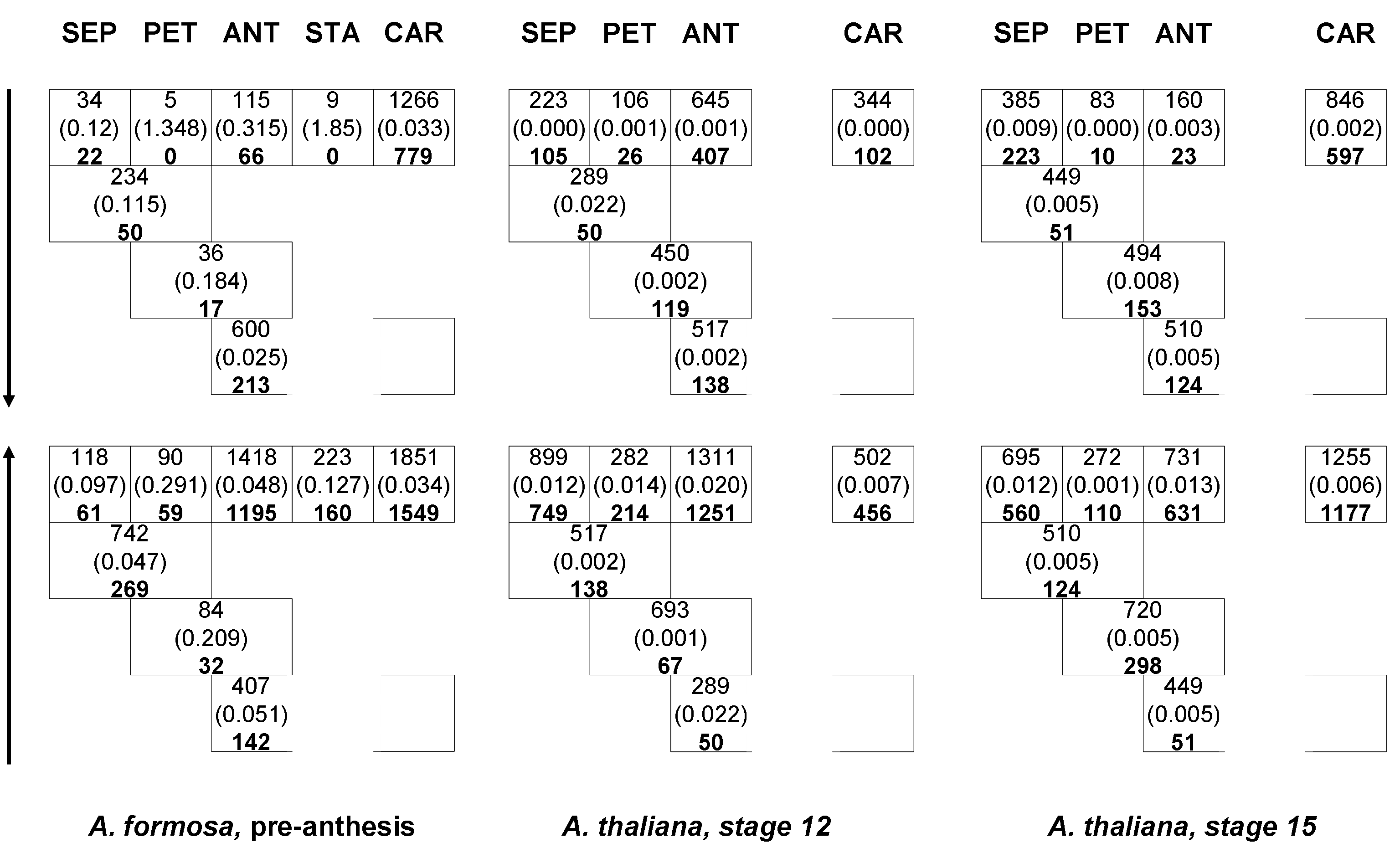

Supplement: Figure S1 — Differentially expressed genes in A. formosa (pre-anthesis) and A. thaliana (stages 12 and 15) flowers. Each square represents one contrast and reports the number of differentially genes, the corresponding false discovery rate as determined by bootstrap analysis in brackets and the number of differentially expressed genes adjusted for genes with higher D statistics with other contrasts. Upper and lower panel depict numbers for down- and up-regulated genes, respectively. (5.74 MB TIF) [file pone.0009735.s001.tif]
