# Supplementary material for: Within and between Whorls: Comparative Transcriptional Profiling of Aquilegia and Arabidopsis
Source: PLoS One. 2010 Mar 23;5(3):e9735. doi: 10.1371/journal.pone.0009735 (PMC2843724; doi:10.1371/journal.pone.0009735)
Supplement: Table S1 — Spearman's rank correlation coefficients of array-wide expression of all pair wise combinations of whorls (* denotes p<0.001). In Aquilegia formosa (AF), most correlations are negative, except for a positive correlation of sepals and petals and no correlation between staminodia and sepals. In Arabidopsis thaliana (AT, stage 12), most correlations are also negative, except for a positive correlation between carpels and petals. (0.05 MB DOC) [file pone.0009735.s002.doc]

**Table S1** Spearman’s rank correlation coefficients of array-wide expression of all pair wise combinations of whorls (* denotes p <0.001). In *Aquilegia formosa* (AF)*,* most correlations are negative, except for a positive correlation of sepals and petals and no correlation between staminodia and sepals. In *Arabidopsis thaliana* (AT, stage 12), most correlations are also negative, except for a positive correlation between carpels and petals.
